# Three-dimensional analysis of spawning behaviour of skipjack tuna *Katsuwonus pelamis* using stereo-image measurements

**DOI:** 10.1242/bio.062671

**Published:** 2026-07-28

**Authors:** Hiroto Yamamoto, Akira Sasaki, Tomoki Kanna, Yasushi Mitsunaga, Shinsuke Torisawa

**Affiliations:** ^1^ Department of Fisheries, Graduate School of Agriculture, Kindai University, 3327-204 Nakamachi, Nara 631-8505, Nara, Japan; ^2^ Exhibition Department, Io World Kagoshima City Aquarium, 3-1 Honkoushinmachi, Kagoshima 892-0814, Kagoshima, Japan; ^3^ Department of Fisheries, Faculty of Agriculture, Kindai University, 3327-204 Nakamachi, Nara 631-8505, Nara, Japan

**Keywords:** Group spawning, Pelagic fish, Multi-stereo camera, Swimming kinematics

## Abstract

Although many pelagic fish species exhibit group spawning, the mechanisms underlying this mating system remain unclear; more fundamentally, detailed descriptions of group-spawning behaviour itself are lacking. Here, the spawning behaviour of skipjack tuna, *Katsuwonus pelamis*, characterised by ‘group spawning’, was recorded in an aquarium tank using stereo-image measurements to estimate the size of reproductive participants and quantify swimming kinematics during reproduction. Events were observed in which an individual (referred to as the ‘leader’) was chased by multiple conspecifics (referred to as the ‘follower’) and subsequently engaged in spawning. However, leaders (presumed female) were rarely chased by multiple followers (presumed male), a scenario more conducive to visible sperm release. In contrast, single followers chased leaders of varying body sizes, including smaller and likely immature individuals. Furthermore, leaders chased by multiple followers darted and turned at higher speeds. Reproduction may begin with one male pursuing one female; when the male targets a female with a high likelihood of spawning, the female accelerates, potentially attracting additional males to join the chase. By linking locomotor performance to reproductive outcomes, this study advances understanding of group-spawning mechanisms in pelagic fish species and contributes to broader theories of sexual selection and mating system evolution.

## Teaser

**Summary:** This study reveals how skipjack tuna individuals spawn in groups, showing how body size and swimming speed shape group-spawning mechanisms.

## INTRODUCTION

When one sex invests more heavily in offspring than the other, the less-investing sex competes for access to the more-investing sex (Trivers, 1972). Specifically, when females invest more than males, males are expected to compete for mates, whereas females preferentially select males that offer superior resources and genetic quality (Davies et al., 2012). In many animals, male reproductive success is strongly influenced by male–male competition for access to females, as classically demonstrated in red deer, where dominant males monopolise harems and achieve disproportionately high mating success (Clutton-Brock et al., 1982). Birds exhibit elaborate vocal displays and possess conspicuous ornamental plumage for attracting mates (Andersson, 1982; Petrie et al., 1991; Partan et al., 2005).

Numerous studies have investigated sexual selection and mating systems in fish, enabled by the practicality of conducting experiments and observations in aquaria (Borghezan et al., 2019; Dosen and Montgomerie, 2004; Karino et al., 2011; Laubu et al., 2016; Makiguchi et al., 2016; Seki et al., 2023; TerMarsch and Ward, 2020; Werner and Lotem, 2003; Yokoi et al., 2015; Zempo et al., 2021). In fish, larger females tend to produce more eggs, leading to the expectation that males may preferentially select larger females to maximise their reproductive success. This preference has indeed been observed across multiple fish species (Dosen and Montgomerie, 2004; Seki et al., 2023; Werner and Lotem, 2003). For example, Werner and Lotem (2003) experimentally demonstrated that males of a cichlid species, *Astatotilapia flaviijosephi*, engaged in longer courtship displays towards larger females. Similarly, Seki et al. (2023) revealed that male chum salmon, *Oncorhynchus keta*, quiver their bodies closer to larger females, a distinct form of courtship behaviour. In some cases, not only male but also female mate choice matters. Females select mates based on signals from males, including visual and tactile cues. For instance, Karino et al. (2011) found that female guppies, *Poecilia reticulata*, prefer males with elongated dorsal fins, despite the potential swimming disadvantage posed by these fins. In terms of tactile cues, TerMarsch and Ward (2020) demonstrated that female fathead minnows, *Pimephales promelas*, adjusted their mate preferences in response to the intensity of male courtship, as detected through their lateral line systems.

Fish species exhibit diverse reproductive strategies, with ‘group spawning’ being particularly prevalent. This mating system involves multiple males chasing a single female immediately before sperm release, which coincides with egg release. This phenomenon has been reported in numerous pelagic fish species (e.g. purplish amberjack, *Seriola dumerili*: Tachihara et al., 1993; Atlantic bluefin tuna, *Thunnus thynnus*: Aranda et al., 2013; and chub mackerel, *Scomber japonicus*: Tsuda et al., 2014). Nevertheless, their reproductive mechanisms remain largely unexplored because of the complexities of group spawning, which involves numerous individuals and typically occurs in the difficult-to-observe open ocean. Tsuda et al. (2014) recorded the spawning behaviour of chub mackerel, *S. japonicus*, in a net cage through video recording, showing that two or three fish break away from the school at high speed, circle tightly to release eggs and sperm, and ultimately return to the school. These rapid swimming and turning behaviours may reflect underlying sexual dynamics in group-spawning species, potentially involving mate preferences and behavioural interactions between reproductive participants. Although the few studies on group spawning have described its outward characteristics, more detailed and quantitative descriptions – such as the number of individuals participating in reproduction, the types of individuals involved, and the processes leading to egg and sperm release – have not been conducted, despite their potential to contribute to elucidating its reproductive mechanisms of group spawning.

Kagoshima City Aquarium is a unique facility that exhibits skipjack tuna, *Katsuwonus pelamis*, a species recognised for its group-spawning behaviour. This facility allows continuous observation of skipjack tuna reproduction and the acquisition of fertilised eggs (Fujioka et al., 2024). Skipjack tuna is a highly migratory species widely distributed throughout the Western and Central Pacific Ocean (WCPO), ranging from 44°N to 37°S (Matsumoto et al., 1984; Wild and Hampton, 1994). In 2019, the annual catch of skipjack tuna in the WCPO exceeded 2.04 million tons (WCPFC, 2023, https://www.wcpfc.int/doc/wcpfc-tuna-fishery-yearbook2022), making it by far the most abundant and commercially important tuna species in the region. The species is highly social and is known to form large mixed-sex schools consisting of up to tens of thousands of individuals. Studies of reproductive ecology in subtropical and temperate waters of the Western Pacific have reported sex ratios ranging from 0.50 to 0.53 (Ashida, 2020), suggesting that spawning groups are likely formed from populations with relatively balanced sex ratios. Larval surveys have indicated that skipjack tuna spawns in waters with sea surface temperatures above 24°C (Matsumoto et al., 1984; Nishikawa et al., 1985; Wild and Hampton, 1994). In the Western and Central Pacific, identified as the main spawning grounds, larvae are observed year-round (Ueyanagi, 1969; Nishikawa et al., 1985). Histological analyses of gonads further confirm that skipjack tuna spawns continuously throughout the year (Ashida et al., 2007, 2008, 2010). Therefore, by optimising environmental conditions, such as water temperature, skipjack tuna spawning can be continuously induced.

Stereo-image measurements represent an effective method for investigating the reproductive mechanisms underlying group spawning. This technique estimates three-dimensional (3D) spatial coordinates in real space by utilising images captured from multiple cameras based on triangulation. It enables non-invasive measurement of fish body length underwater (Harvey et al., 2002; Torisawa et al., 2011; Ikeya et al., 2022; Yamamoto et al., 2025) and facilitates analysis of swimming behaviour and turning performance (Fish et al., 2018; Harada et al., 2021).

In this study, we aimed to provide the first detailed description of skipjack tuna reproductive behaviour, with particular focus on swimming speed, turning performance, and the body size of individuals involved in spawning events. Body size may help elucidate the characteristics of reproductive participants, including their maturity status and patterns of mate preference. Swimming speed and turning performance are expected to be important factors for males, as rapid movement may increase their ability to fertilise eggs immediately after they are released by females. To accomplish this, we employed stereo-image measurements to non-invasively quantify body size and kinematic traits during reproduction and confirmed visible sperm release. Although sperm release does not necessarily guarantee reproductive success, it is one of the important prerequisites for reproduction. We hypothesised that, if leaders correspond to females, larger individuals would be followed more frequently, suggesting size-related mate preferences. In addition, we predicted that rapid swimming and tight turning performance could be important factors for followers (presumed male), potentially enabling them to achieve sperm release more successfully; accordingly, sperm release was expected to be associated with faster spawning events. The findings of this study are expected to advance understanding of the mechanisms underlying group spawning in pelagic fishes, contribute to broader theories of sexual selection and mate choice, and inform management and conservation of tuna populations, which hold considerable ecological and economic importance.

## RESULTS

### Characteristics of reproductive behaviour

**Fig. 1.**
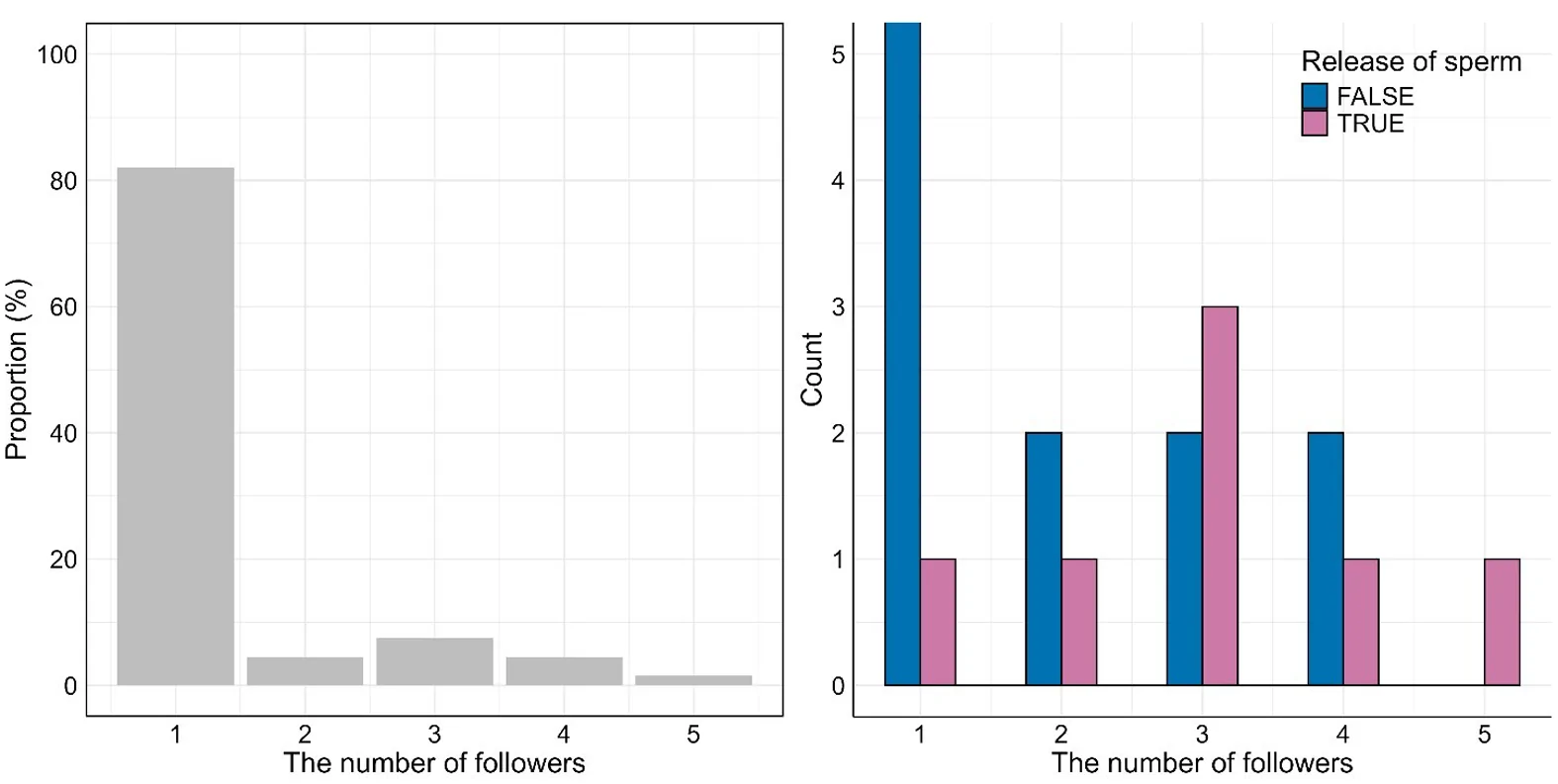
**Characteristics of reproductive behaviours in skipjack tuna.** The left panel shows proportions of the number of followers in observed reproductive behavioural events. The right panel shows the frequency of reproductive behavioural events with (magenta bar) and without (blue bar) sperm release.

The number of followers ranged from one to five (Fig. 1). However, single-follow events constituted more than 80% of the recorded reproductive behaviour events. Out of the 85 reproductive behavioural events observed, spawning was confirmed in seven cases. Spawning occurred once in 65 single-follow events and six times in 20 plural-follow events (Movie 1). As shown in Movie 1, the individual closest to the leader released sperm, suggesting that the leader and the chasing individuals were likely to be presumed female and presumed male, respectively. The proportion of spawning differed significantly between single- and plural-follow events (*P*<0.001). Spawning events occurred at 6 min 4 s, 13 min 19 s, 24 min 0 s, 38 min 12 s, 50 min 23 s, 51 min 11 s, and 52 min 23 s after the start of recording.

### Fork length of reproductive participants

**Fig. 2.**
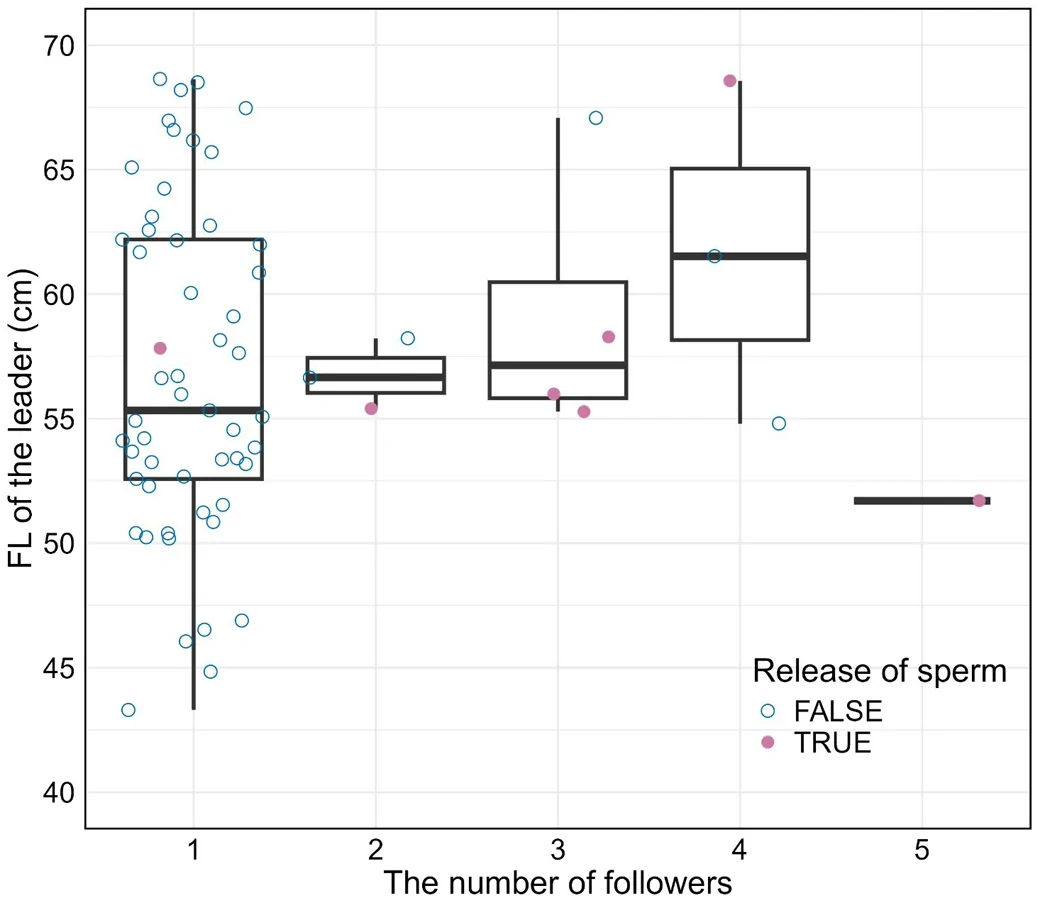
**Relationship between the number of followers and fork length of the leader.** Each box shows the fork length of leaders followed by one (*n*=55), two (*n*=3), three (*n*=5), four (*n*=3), and five followers (*n*=1). Magenta filled circles and blue open circles indicate whether sperm release was observed or not, respectively.

**Fig. 3.**
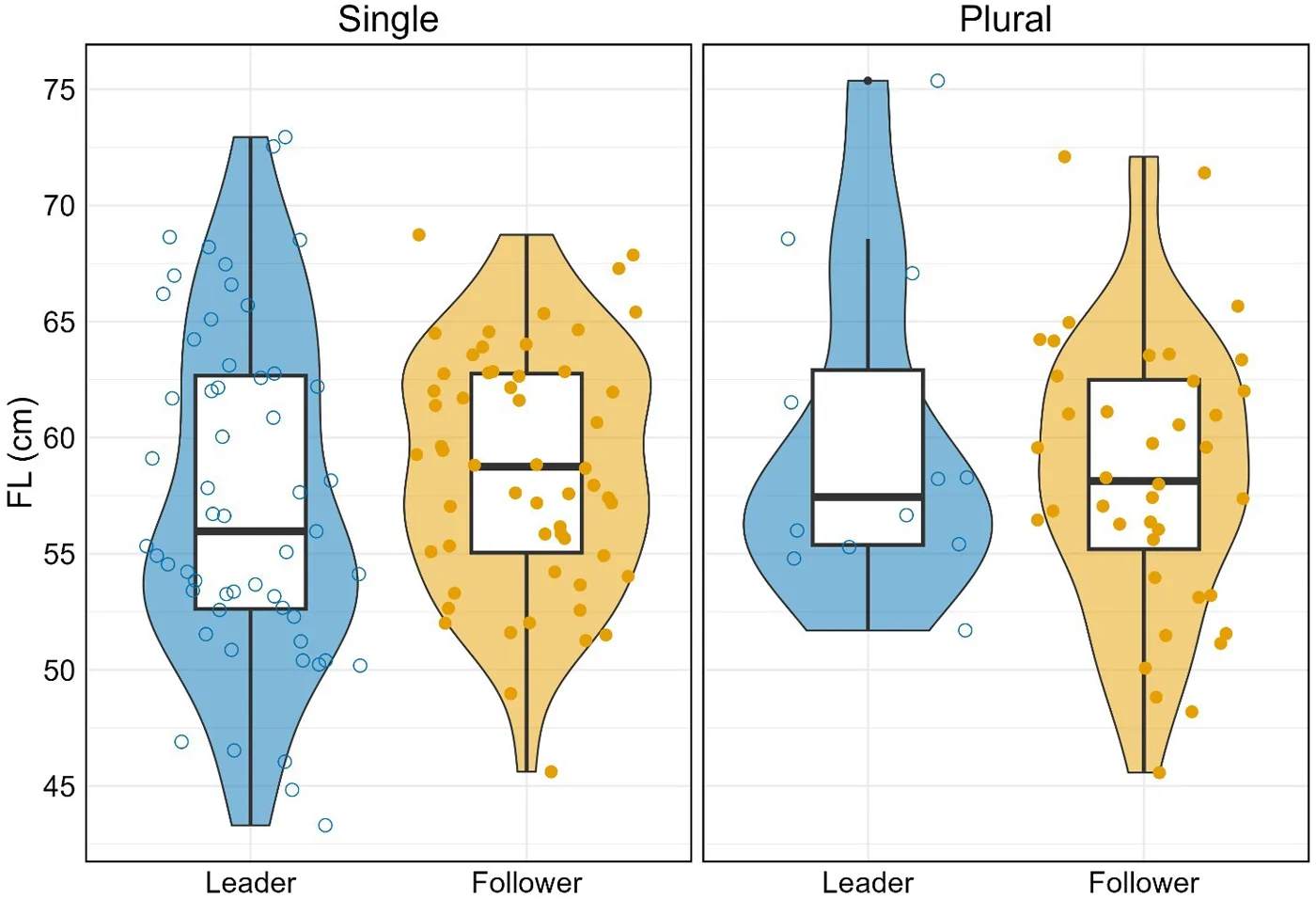
**Fork lengths of the leader and followers in single- and plural-follow events.** Each box indicates the respective fork lengths of the leader and followers in single-follow (left panel) and plural-follow (right panel) events. Blue open circles and orange filled circles show the raw data of fork lengths of the leader and followers, respectively. There was no significant difference between the fork lengths of leaders and followers for single- and plural-follow events. In addition, the fork length distributions of leaders and followers exhibited no significant differences in both single- and plural-follow events.

No significant correlation was found between the number of followers and fork length of the leader (Fig. 2; ρ=0.15, *P*=0.24). The fork lengths of leaders in single-follow events ranged widely from 43.3 to 72.9 cm. When comparing the fork lengths of leaders and followers for single- and plural-follow events (Fig. 3), no significant differences were observed (single-follow event: *W*=1340, *P*=0.24; plural-follow event: *W*=651, *P*=0.57). The fork length distributions of leaders and followers exhibited no significant differences in both single- and plural-follow events (Fig. 3; single-follow event: *D*=0.22, *P*=0.11; plural-follow event: *D*=0.15, *P*=0.87). Furthermore, the fork length of the leader was 57.8 cm (*n*=1) in single-follow events and 57.5±5.8 cm (mean±s.d., *n*=6) in plural-follow events related to spawning behaviour. The occurrence of sperm release was strongly affected by follow type (single or plural), whereas leader body size had no significant effect (follow type: *z*=−3.43, *P*<0.001, the fork lengths of leaders: *z*=−0.99, *P*=0.32).

### Differences in reproductive behaviour based on the number of followers

**Fig. 4.**
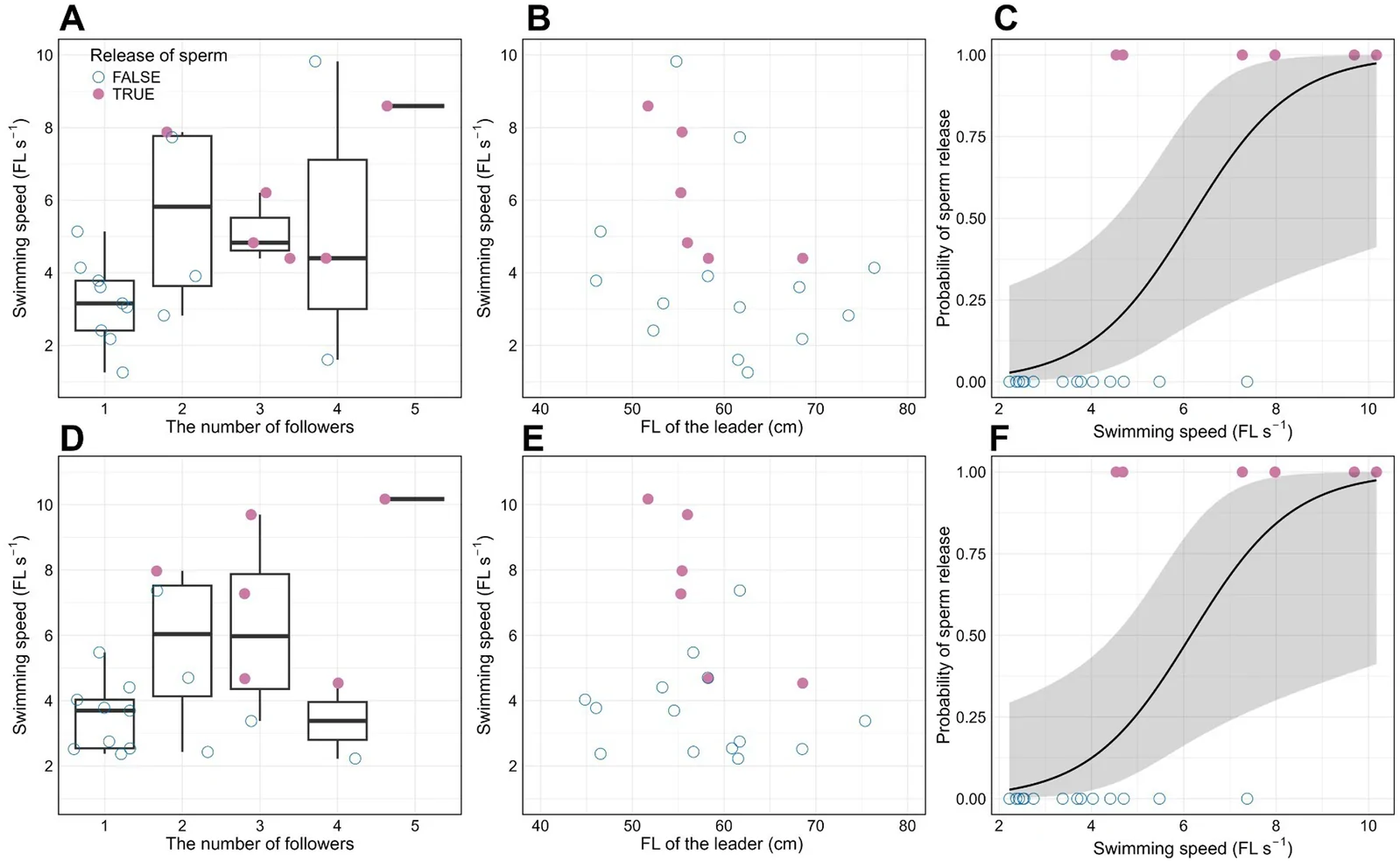
**Relationship between the swimming speed of the leader and the number of followers, fork length of the leader, and probability of sperm release.** The top three panels show the relationships during straight swimming (A–C), whereas the bottom three panels show those during turning (D–F). Magenta filled circles and blue open circles indicate whether sperm release was observed or not, respectively.

The leader darted at speeds ranging from a minimum of 1.3 FL s^−1^ with one follower to a maximum of 9.8 FL s^−1^ with four followers (Fig. 4A). A positive correlation was observed between the number of followers and swimming speed of the leader (Fig. 4A; *t*=2.6, d.f.=18, *P*=0.020, *r*=0.52). No significant difference in swimming speed was found among varying numbers of followers (Fig. 4A; *H*=6.6, d.f.=4, *P*=0.16). The swimming speed of leaders during spawning behavioural events was 6.1±1.8 FL s^−1^ (*n*=6), while that during single-follow events without sperm release was 3.9±2.3 FL s^−1^ (*n*=14), suggesting that leaders swam faster during spawning behavioural events (Fig. 4B; *W*=13, *P*=0.02). For plural-follow events where spawning was not observed, the swimming speed of the leader was 5.2±3.5 FL s^−1^ (*n*=5). The probability of sperm release tended to increase with swimming velocity (Fig. 4C; *z*=1.74, *P*=0.08).

**Fig. 5.**
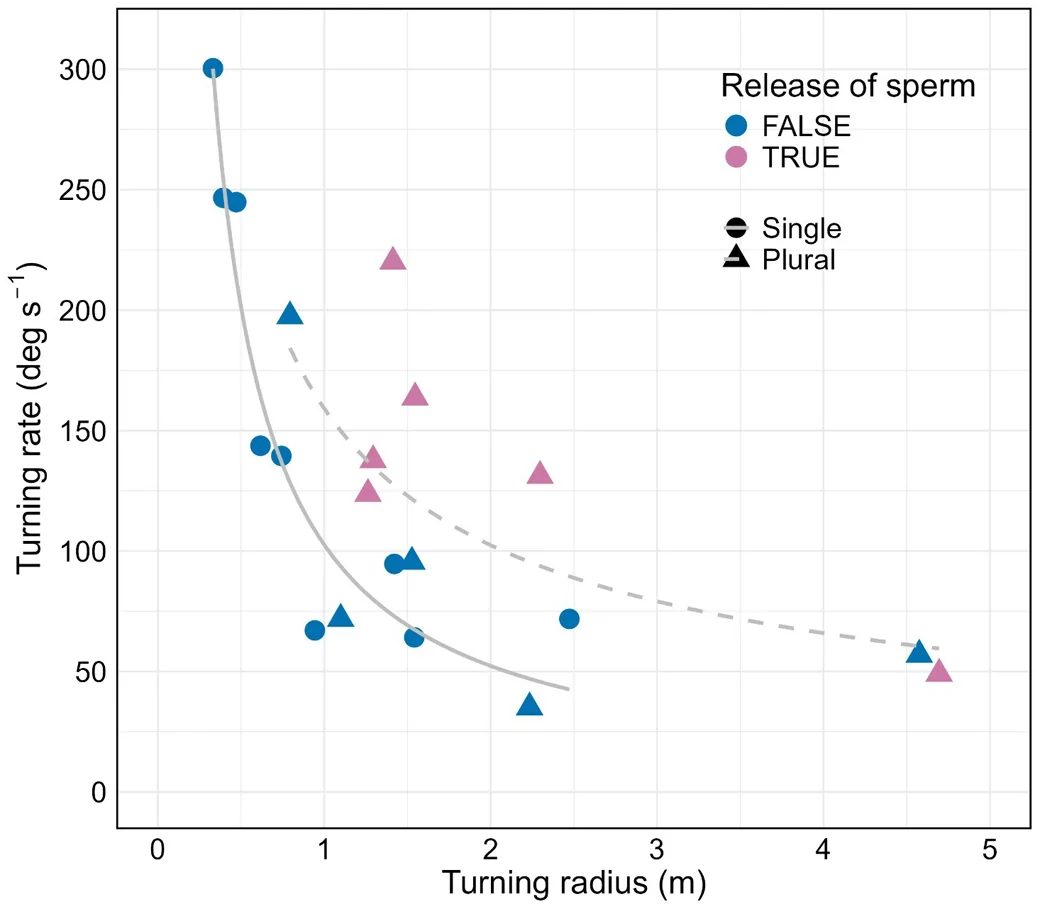
**Relationship between turning radius and rate in single- and plural-follow events.** Circles and triangles represent single- and plural-follow events, respectively, while solid and dotted lines denote each approximate curve; ω=103*R*^−1.0^ (single-follow event) and ω=159*R*^−0.6^ (plural-follow event). Magenta and blue colours indicate cases with and without sperm release.

A positive correlation was found between the number of followers and swimming speed during turning (Fig. 4D; *t*=2.3, d.f.=18, *P*=0.032, *r*=0.48); however, no significant difference in swimming speed was detected across varying numbers of followers (Fig. 4D; *H*=7.3, d.f.=4, *P*=0.12). In comparing the relationship between turning radius and rate for single- and plural-follow events (Fig. 5), the turning rate for plural-follow events was higher than that for single-follow events at equivalent turning radius values. The relationship between turning radius and turning rate appears to differ between single- and plural-follow events; however, this pattern is likely driven largely by two cases in plural-follow events with a turning radius of approximately 4.5 m (Fig. 5). The turning radius of the leaders during spawning behavioural events was 2.1±1.3 m (*n*=6), while that during following behavioural events was 1.4±1.1 m (*n*=14). For plural-follow events without confirmed spawning, the turning radius was 2.1±1.5 m (*n*=5). The swimming speed of leaders during turns in spawning behavioural events was 5.9±2.8 FL s^−1^ (*n*=6). The turning speeds during plural- and single-follow events without spawning were 4.7±2.3 FL s^−1^ (*n*=5) and 3.5±1.1 FL s^−1^ (*n*=9), respectively, thereby indicating that leaders exhibited rapid turning movements during spawning behavioural events (Fig. 4E; *W*=7, *P*=0.002). In fact, the probability of sperm release significantly increased with swimming velocity during turning (Fig. 4F; *z*=2.33, *P*=0.02).

## DISCUSSION

To the best of our knowledge, this study is the first to document the reproductive behaviour of skipjack tuna. The frequency of plural-follow events, as indicated by previous studies (e.g. Tsuda et al., 2014), was lower than that of single-follow events, which were substantially more prevalent, accounting for more than 80% of all recorded reproductive behavioural events (Fig. 1). However, only one out of 65 single-follow events confirmed sperm release, whereas six out of 20 plural-follow events did so. This suggests that plural-follow events are associated with visible sperm release. Although sperm release is not equal to reproductive success, considering that fertilised eggs were obtained during recording and that sperm release was not observed in single-follow events, plural-follow events are more likely to be associated with reproductive success. Males may respond to females exhibiting a high likelihood of egg release, resulting in sequential chasing that ultimately culminates in plural-follow events. In events involving visible sperm release, chasing interactions may last longer and become more dynamic, raising the possibility that egg and sperm release occur before additional individuals join the chase. If sperm release occurred outside the camera's field of view and only the subsequent participation of additional followers was recorded, the relationship between sperm release and plural-follow events may have been underestimated. Hormones secreted by females are recognised for their role in inducing reproductive behaviour in males across various fish species (Moore and Waring, 1996; Pradhan and Olsson, 2015; Yabuki et al., 2016). Furthermore, male salmonids exhibit changes in courtship behaviour as females approach egg release (Esteve, 2005; Seki et al., 2023). Male skipjack tuna individuals may also sense the timing of egg release through various cues, such as hormones released by females. However, because the current study did not confirm that the leader of the spawning group was female and the followers were male, we cannot exclude the possibility that excited individuals were chasing or attempting to attack same-sex conspecifics or that multiple males and females were present within a spawning group.

In the northern habitat limit of the North-Western Pacific Ocean, the spawning potential group of skipjack tuna migrates towards tropical and subtropical areas, whereas the residence group resides around the northern habitat for feeding and avoiding unfavourable environments (Ueda et al., 2025). The occurrence of immature individuals within spawning potential groups can be lower than that of the current study. If immature individuals are less likely to attract multiple followers, environments with a greater abundance of reproductively competent individuals in the wild may promote more frequent following by multiple individuals, potentially leading to a higher occurrence of plural-follow events.

Furthermore, a quantitative assessment was performed on the body size of individuals participating in reproduction and swimming characteristics associated with reproductive behaviour (e.g. swimming speed and turning performance) using stereo-image measurements. Estimating the body size of individuals involved in reproduction not only provides insights into size-assortative mate preferences during group spawning but also offers direct evidence for reproductive ecology studies, which have traditionally relied on histological examinations of gonads. Swimming characteristics during reproduction, such as swimming speed and trajectories, are essential for males, as they need to swim near females to fertilise the eggs they release.

### Fork length of reproductive participants

The leaders in single-follow events ranged widely in size from a minimum of 43.3 cm to a maximum of 72.9 cm (Figs 2 and 3). The confirmation of sperm release occurring only once during single-follow events suggests that immature individuals may have been chased. Ashida (2020) estimated the fork length to be 55.6 cm for female skipjack tuna individuals at 50% maturity in temperate regions (25–45°N). Based on this, the individuals most frequently chased during single-follow events (52–55 cm) likely had a maturity rate below 50%. In addition, a potential factor is the spawning frequency (spawning interval). The spawning frequency of skipjack tuna individuals in temperate regions (25–45°N) is 0.48 times per day (spawning interval of 2.10 days; Ashida, 2020). This suggests that the skipjack tuna individuals examined in this study may have recently spawned. However, spawning frequency (confirmed sperm release) within 1 h appears relatively high. The fork length of the leader did not relate to the number of followers (Fig. 2). In addition, the high frequency of single-follow events and diversity of leader sizes (including smaller individuals) indicate that followers (presumed male) do not preferentially chase larger leaders (presumed female), despite their higher maturity rates and greater batch fecundity (Ashida, 2020; Ashida et al., 2007, 2008, 2017). Because neither the sex nor the identity of reproductive participants could be determined in this study, we cannot exclude the possibility that the same individual was repeatedly followed and attracted multiple individuals across spawning events. The ability to track and identify individuals across events would facilitate a better understanding of sex-specific mate preferences in group spawning. Instead of preferentially following larger leaders, presumed males may chase indiscriminately to maximise reproductive opportunities by positioning themselves closer to the female, regardless of body size. Size-assortative mating has been frequently reported in pair-spawning and semelparous species (Dosen and Montgomerie, 2004; Seki et al., 2023; Werner and Lotem, 2003; Karino et al., 2011; TerMarsch and Ward, 2020), where each reproductive event is likely to have a strong influence on individual fitness. In contrast, in iteroparous species such as many pelagic fishes, the greater number of reproductive opportunities may weaken size-based mate preferences.

### Differences in reproductive behaviour based on the number of followers

The swimming speed positively correlates with the number of followers (Fig. 4A). The swimming speed was lowest at 1.3 FL s^−1^ with one follower and highest at 9.8 FL s^−1^ with four followers. This finding aligns with that of previous research that estimated the swimming speed range of skipjack tuna via video analysis (0.5–14.4 BL s^−1^; Yuen, 1966). In the wild, unusually elevated body temperatures have been reported exclusively in spawning potential groups and have been hypothesised to reflect spawning behaviour (Ueda et al., 2025). The high swimming speeds observed during reproductive events in this study would likely require high-frequency caudal fin movements, which could contribute to the elevated body temperatures observed in wild fish. However, these elevated speeds are likely achievable only for short durations. Because such intense swimming activity may lead to overheating, wild individuals may subsequently dive into deeper, cooler waters as a form of behavioural thermoregulation to dissipate excess body heat (Holland et al., 1992; Fujioka et al., 2021).

In terms of turning performance, individuals engaged in plural-follow events exhibited larger curvature radii than those in single-follow events (Fig. 5). This finding aligns with that of previous research, which indicates that curvature radius increases with swimming speed (Fish and Nicastro, 2003; Fish et al., 2018), particularly as swimming speed increases with the number of followers (Fig. 4D). Furthermore, an inverse relationship between turning radius and rate was observed (Fig. 5). This trend is consistent with that of other species (e.g. whirligig beetle, *Dineutes horni*: Fish and Nicastro, 2003; batoid rays: Parson et al., 2011; manta, *Mobula birostris*: Fish et al., 2018; and Pacific bluefin tuna, *Thunnus orientalis*: Downs et al., 2023). The turning rates of leaders in plural-follow events were higher than those in single-follow events at equivalent turning radii. This suggests that the leaders chased by multiple followers swam faster in both straight-line and circular swimming (Fig. 4A,D). Moreover, the turning radius of leaders during plural-follow events exhibited no significant difference between spawning (2.1±1.3 m, *n*=6) and non-spawning (2.1±1.5 m, *n*=5) cases. However, the turning rate of leaders during non-spawning plural-follow events was 91.4±63.2 deg s^−1^ (*n*=5), whereas it was significantly higher at 138±55.8 deg s^−1^ (*n*=6) during spawning-confirmed plural-follow events. This suggests that leaders swam faster and performed tighter turns during spawning events.

By utilising stereo-image measurements, we were able to quantitatively evaluate swimming performance, including swimming speed and turning ability, during reproductive events in this species. However, the study had several limitations. Capturing the entire sequence with cameras proved challenging, thereby limiting our analysis to localised observations due to the dynamic nature of spawning behaviour (Movie 1). Furthermore, the results obtained in this study, viewed in isolation, are insufficient to comprehensively discuss reproductive strategies in group spawning. In particular, the inability to identify the sex of reproductive participants and to verify fertilisation success for each reproductive event represents a major obstacle to understanding sex-specific roles and the mechanisms underlying reproductive success. To address this limitation, it is necessary to identify the individual fish participating in reproduction and investigate the positional relationship within the spawning group of males responsible for fertilising the eggs obtained during the event. In addition, because this study examined spawning events under captive conditions (e.g. higher population density and shorter inter-individual distances than in the wild due to the tank capacity and the existence of other species), it cannot be ruled out that a higher proportion of events, such as single-follow events, did not lead to successful spawning. The captive population consisted of only 36 individuals, which is likely to be a smaller shoal in the wild. Therefore, the relatively small group size may have reduced the number of reproductively active individuals available to participate in spawning events and influenced the frequency of plural-follow behaviour observed in this study. Because population size, population density, and operational sex ratio can affect mating interactions, future studies should investigate how these factors shape spawning behaviour in natural populations.

This study revealed that reproductive events involving multiple followers, associated with a higher likelihood of visible sperm release, infrequently occur during group spawning. In contrast, single-follow events, which were overwhelmingly more frequent, involved the pursuit of individuals exhibiting a lower likelihood of maturity. This potentially contributed to the low number of cases leading to fertilisation. Stereo-image measurements were used to estimate the body size of individuals participating in reproduction, indicating that presumed males do not preferentially chase a presumed female. Reproduction may be initiated with a single follower chasing a single leader (including smaller individuals); as additional followers participate, the process ultimately leads to spawning and fertilisation. Additionally, estimates of swimming speeds and turning performances during reproduction suggest that reproductive events involving sperm release are associated with faster swimming and turning capabilities. This speed is considered critical for males because it allows them to rapidly fertilise the eggs released by females, thereby ensuring their reproductive success. Future research that combines individual identification with paternity analysis of fertilised eggs is essential for enhancing our understanding of reproductive strategies in group-spawning contexts.

## MATERIALS AND METHODS

### Experimental site and rearing conditions

The recording occurred on 7 September 2022 in the indoor water tank (24 m×13 m, 5 m depth) at the Kagoshima City Aquarium. The tank contained no physical structures other than a curtain installed to prevent fish from colliding with the tank walls. In addition to skipjack tuna, several other species were present in the tank during the study period, including whale shark (*Rhincodon typus*), spotted eagle ray (*Aetobatus narinari*), Indian mackerel (*Rastrelliger kanagurta*), kawakawa (*Euthynnus affinis*), and yellowfin tuna (*Thunnus albacares*). Skipjack tuna individuals, captured through trolling in the East China Sea off Kagoshima Prefecture, were kept in captivity for approximately 1 to 3 years. The number of individuals was monitored monthly by staff, and 36 skipjack tuna were present in the tank at the time of recording. However, the sex ratio of the individuals was unknown. Seawater was pumped from the nearby coastal area and maintained at approximately 25°C throughout the year using heaters. Fish in the tank were fed once daily at 10:15. The diet consisted primarily of mackerel and krill.

### Stereo-video recording procedures

**Fig. 6.**
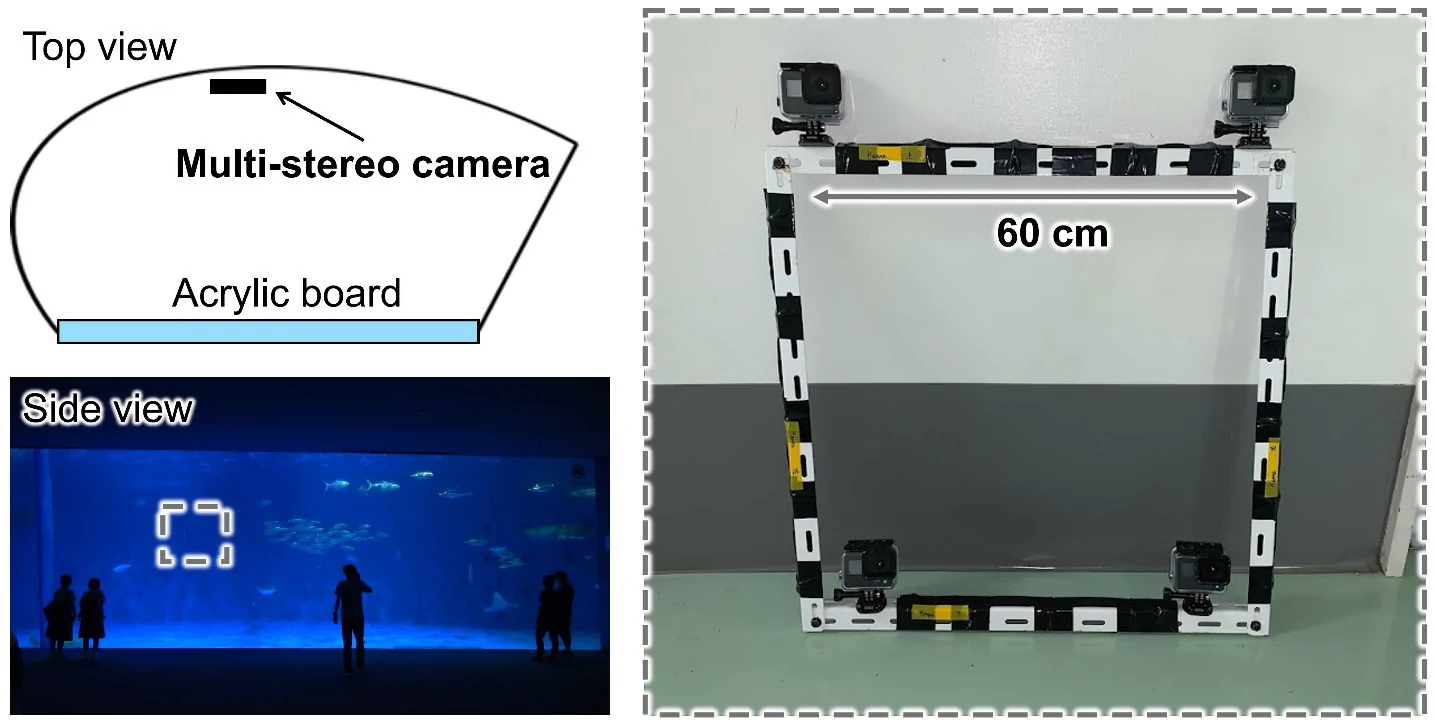
**Stereo camera used in this study and the filming set-up.** The left panels show schematic views of the tank from the top and from the side, respectively. The right panel shows a multi-stereo camera that comprises four cameras. Each camera is approximately 60 cm apart. The multi-stereo camera system recorded reproductive behaviour occurring in the centre of the tank.

Preliminary observations indicated that reproductive behaviour began around 16:00 and ended within 1 h. In addition, the fertilised eggs were collected during that time. Therefore, we conducted video recordings for approximately 1 h from 16:00. Four identical waterproof cameras (GoPro HERO9, GoPro Inc., CA, USA) were mounted to the corners of a metal frame to create a multi-stereo camera system (Fig. 6). The multi-stereo camera system was anchored to the tank with ropes so that it captured the centre of the tank, as well as both the water surface and tank bottom (Fig. 6). To synchronise all cameras, a flashing light was recorded prior to recording the target. The recording was performed using a wide field of view, a resolution of 1920×1080 pixels, and a frame rate of 30 fps. When set to a wide-angle setting, the edges of the field of view are distorted in air; however, underwater, the field of view becomes effectively linear due to light refraction. A robust 3D frame (120 cm per side) was photographed prior to the recording for image calibration. A sampling net was anchored to the water surface to collect fertilised eggs during the recording. The collected fertilised eggs were confirmed to be in the early developmental stage using a microscope and subsequently frozen in 99% ethanol. The fertilised eggs were used for species identification through next-generation sequencing and then identified as eggs of skipjack tuna. Therefore, it was confirmed that skipjack tuna certainly spawned during the filming period.

### Calibration of images captured by a multi-stereo camera system

Image calibration was performed using a 3D frame (120 cm per side), employing the direct linear transformation (DLT) method (Hartley and Zisserman, 2003; Torisawa et al., 2011; Ikeya et al., 2022; Yamamoto et al., 2025). This method allows for the acquisition of 3D spatial coordinates from images captured simultaneously by multiple cameras. In this process, the spatial and image positions are linked through the internal and external parameters of the camera, which are defined by the focal length, installation position, and orientation. Highly precise and accurate measurements were achieved by photographing a reference 3D frame with calibration points. Calibration enables the calculation of 3D spatial coordinates of image points by applying equations that incorporate DLT parameters, which are established via the least squares method, as expressed below:
(1)

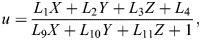

(2)

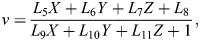
where *u* and *v* are the *x* and *y* coordinates of a point in the 2D coordinate system, respectively; *X*, *Y*, and *Z* are the respective positions in the 3D coordinate system; *L*_1_ to *L*_11_ are the DLT parameters corresponding to each camera. In this study, eight calibration points were distributed within a 3D frame, which were subsequently captured underwater for image calibration. The 3D spatial coordinates of each calibration point were measured using a tape measure and utilised as the true positions. Image calibration was performed using 3D motion analysis software (Move-tr/3D, Library Co.) by manually detecting the calibration point coordinates on the images. Calibration derived from eight reference points of the 3D cubic frame, captured from various angles, facilitates the estimation of the 3D spatial coordinates. The mean error and standard deviation of the known and estimated 3D coordinates for the eight reference points were 0.69 cm (<1%)±0.26 cm. Furthermore, our previous study showed that when a target was located more than 10 m from the multi-stereo camera system, its size was overestimated by approximately 10% (Yamamoto et al., 2025). Therefore, only events occurring within 10 m of the camera system were included in the analysis.

### Examination of reproductive processes and characterisation of spawning behaviour

Video footage from one of the four cameras in the multi-stereo camera system was used to analyse the reproductive behaviour of skipjack tuna individuals, documenting the number of individuals involved in chasing (followers). The white cloud released by skipjack tuna during or immediately after chasing was counted as sperm release, which is one of the important prerequisites for reproduction. The results showed that sperm release did not consistently occur, even in instances of chasing (Fig. 1). In this study, events characterised by chasing without sperm release (e.g. unconfirmed sperm release) were categorised as ‘following behaviour’. Events confirming sperm release were categorised as ‘spawning behaviour’, while the combination of following and spawning behavioural events was collectively termed ‘reproductive behaviour’. The leading individual (presumed female) in the spawning group was defined as the ‘leader’, while the other individuals (presumed male) were classified as ‘followers’. In the context of reproductive behaviour, a single-follower event was categorised as ‘single follow’, while a two-or-more-follower event was categorised as ‘plural follow’.

### Video analysis

#### Body size of individuals participating in reproduction

Reproductive behavioural events were analysed from video footage (*n*=85), documenting both the number of followers and presence or absence of sperm release. Moreover, the fork length of individuals participating in reproductive behavioural events was assessed using 3D motion analysis software (Move-tr/3D, Library Co.). To minimise errors in detecting measurement points owing to tail fin vibrations or motion blur, coordinates from five consecutive frames were obtained, and their average was utilised as the representative value.

#### Reproductive behaviour in relation to the number of followers

The straight swimming behaviour observed during reproductive activities was analysed from video footage (*n*=23) and converted into a sequence of images. These 3D position coordinates of the snout tip of the leader (*x*_*n*_, *y*_*n*_, and *z*_*n*_) were extracted from these images using 3D motion analysis software (Move-tr/3D, Library Co.). The swimming speed *V*_*n*_ (cm s^−1^) was calculated from coordinates recorded at 0.1-s intervals, according to the following formula:
(3)

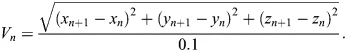
Furthermore, turning swimming behaviour was extracted (*n*=22) and converted into a sequence of images. The 3D position coordinates of the snout tip of the leader were extracted from these images using 3D motion analysis software (Move-tr/3D, Library Co.). The radius *R* (cm) of the curvature circle intersecting more than three points in the turning swimming trajectory was determined using the following formula:
(4)

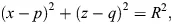
where *p* and *q* are the *x* and *z* coordinates of the centre of the curvature circle, respectively, and *R* (*R*>0) is the turning radius (cm). The turning rate *ω* (rad s^−1^) was calculated using the following formula:
(5)

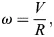
where *ω* is the turning rate (rad s^−1^), *V* is the swimming speed (cm s^−1^), and *R* is the curvature radius (cm). The calculated turning rate *ω* (rad s^−1^) was converted to degrees per second (deg s^−1^) using the following formula:
(6)

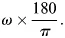


### Statistical analysis

Statistical analysis was conducted using R software (version 4.3.1, R Core Team). Fisher's exact test was used to compare the proportion of spawning between single- and plural-follow events. Spearman's rank correlation coefficient was employed to analyse the relationship between the fork length of the leader and number of followers. The Mann–Whitney *U* test was used to compare the fork lengths of leaders and followers, while the Kolmogorov–Smirnov test was utilised to assess their length distributions. We fitted a generalised linear model (GLM) with a binomial error distribution and logit link function, with sperm release (presence/absence) as the response variable and leader body size and chase type (single versus plural) as explanatory variables. Pearson's correlation coefficient was used to investigate the relationship between swimming speed and number of followers during both straight swimming and turning. The Kruskal–Wallis test was employed to compare swimming speeds in relation to the number of followers during both straight swimming and turning. The Mann–Whitney *U* test was used to compare swimming speeds in relation to sperm release during both straight swimming and turning. We fitted a GLM with a binomial error distribution and logit link function, with sperm release (presence/absence) as the response variable and swimming speeds as explanatory variables during both straight swimming and turning. The significance level was set at 5% for all tests conducted.

## Supplementary Material

10.1242/biolopen.062671_sup1 Supplementary information
