# Supplementary material for: Three-dimensional analysis of spawning behaviour of skipjack tuna Katsuwonus pelamis using stereo-image measurements
Source: Biol Open. 2026 Jul 28;15(7):bio062671. doi: 10.1242/bio.062671 (PMC13488411; doi:10.1242/bio.062671)
Supplement: Supplementary file 1 [file biolopen-15-062671-s1.pdf]

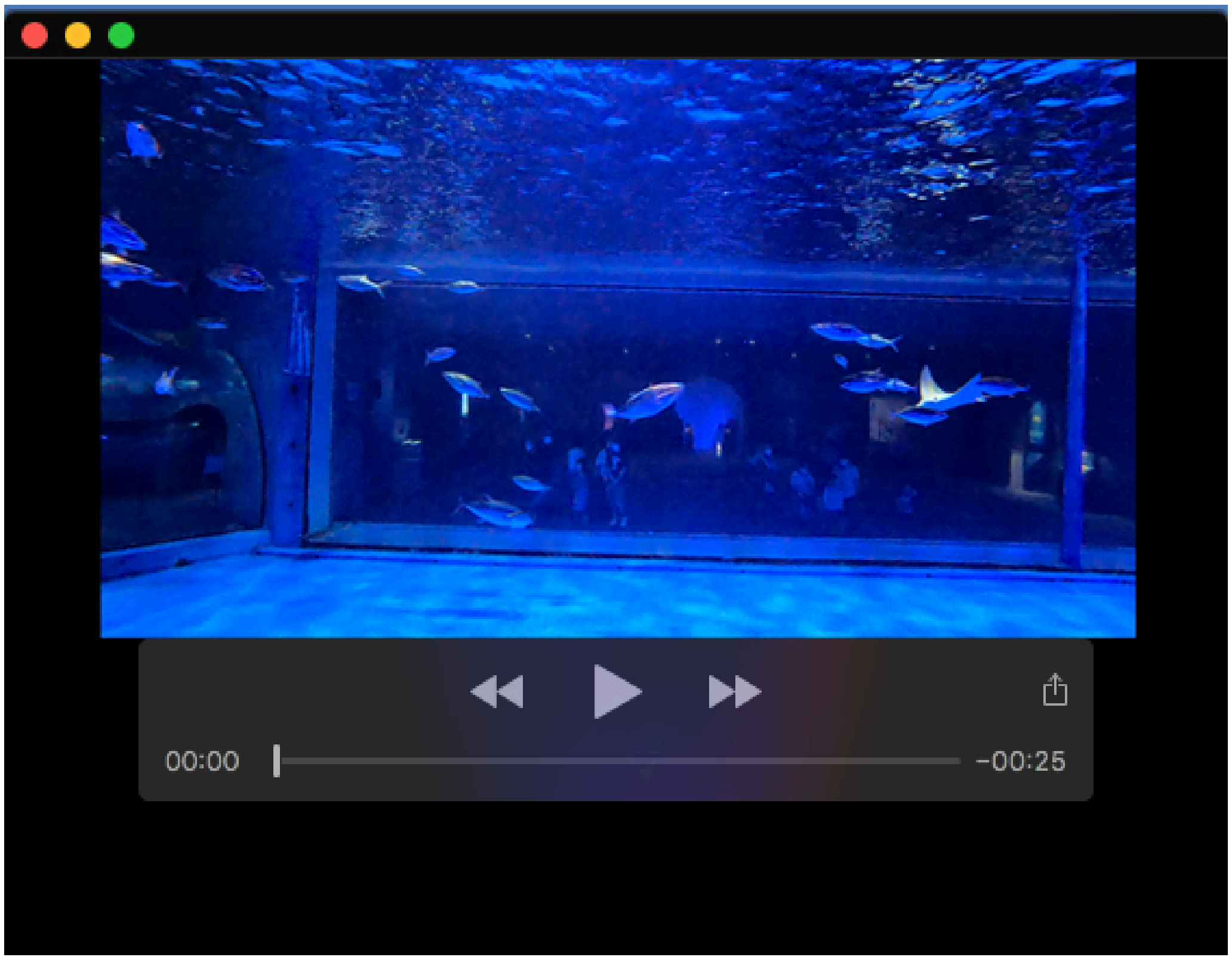

Movie 1.
